# Researchers Look to Snakes’ Evolution to Help Find Treatments for Their Venom

**DOI:** 10.1021/acscentsci.3c00962

**Published:** 2023-08-09

**Authors:** Virat Markandeya

Tucked away in Tamil Nadu, a state at India’s southern tip, is a facility run by members of the Irula tribe, an ethnic group known internationally for a long tradition of snake handling. Because it’s the only large-scale venom extraction facility in India, antivenom manufacturers in the country have depended on the Irula snake catchers cooperative for about 45 years.

But this dependence has created a problem that’s tough to unravel. The cooperative’s venom samples are particular to the region.

Species these snake handlers work with are found in many parts of India, but even within the same species, snakes have developed varying recipes and proportions of toxic proteins in their venoms. Ultimately, this means many antivenoms developed on the basis of samples from Tamil Nadu aren’t as effective against snakebites elsewhere in India, a country with tens of thousands of snakebite-related deaths per year, the highest total in the world.

**Figure:**
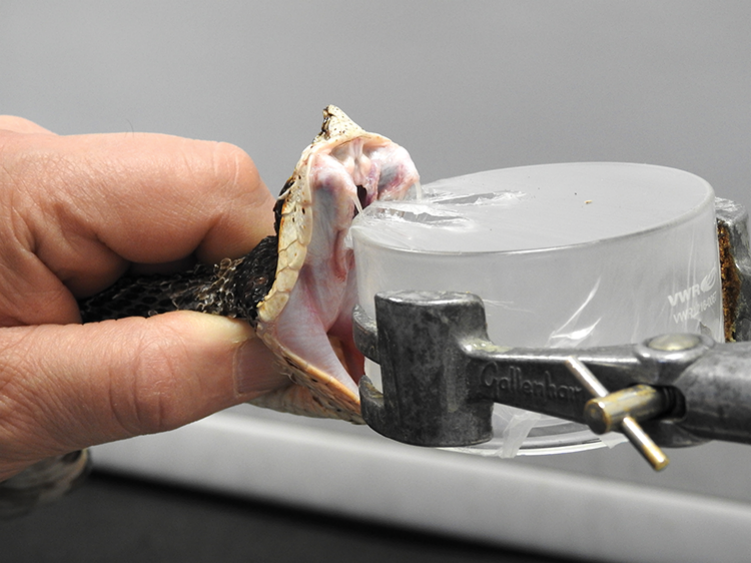
Paul Rowley, a herpetologist at the Liverpool School of Tropical Medicine, draws venom from a Malayan pit viper. The venom is being extracted for research to test how well new therapies inhibit the venom’s activity. Credit: R. Prendergast.

Kartik Sunagar, an evolutionary biologist at the Indian Institute of Science (IISc), is working to bridge the gap in venom availability. Just last July, the foundation was laid for what is to be a first-of-its-kind antivenom research center in Bengaluru, about 340 km away from the center in Tamil Nadu, and Sunagar is one of the scientists leading its setup and development.

A collaboration between the Institute of Bioinformatics and Applied Biotechnology and the IISc, the new research center’s serpentarium is slated to house hundreds of snakes from regions across the country. The center will identify how venoms have diverged, through both changes to DNA across species as well as variations in gene regulation within species.

“It gives us an unparalleled opportunity to have constant access to the venoms, which is just not possible any other way,” Sunagar says. He adds that the facility is going to help not only develop the next generation of Indian antivenoms but also fine-tune current ones.

Evolutionary researchers have leaned into venom profiling to look at the molecules that make up these mixtures. By zooming in on the structures of the proteins and peptides in different snakes’ venoms, they are uncoiling mysteries behind snakes’ defensive adaptations and using that knowledge to work on new toxin-based drugs. And as they sift through the multitudes of venom toxins using modern proteomics—techniques that attempt to catalog all the proteins in a sample—they’re spotting snippets of amino acids that snakes share across species. These common features betray the evolutionary links between these snakes and could be prime targets for drug developers searching for broad-spectrum antivenoms.

## The wide world of toxins

The evolution of snake toxins sped up about 54 million years ago. This happened at the same time that snakes were evolving a high-pressure front fang, like a hypodermic needle that could inject venom into another animal. “They were now able to inject this venom much more effectively,” Sunagar says.

As new species developed, venoms differentiated. Specifically, the amino acids sticking out from the proteins’ surfaces—and thus the cellular receptors they target in their prey—diverged. In another layer of complexity, the venom makeup between regional populations of the same species naturally drifted due to up- or downregulation of certain toxin genes.

In 2022, according to a paper in *Nature Reviews Chemistry*, the UniProt protein database contained almost 3,000 variants of snake toxins. “All of that variation causes us a real headache when we’re trying to make treatments,” says Nicholas Casewell, who heads the Centre for Snakebite Research and Interventions at the Liverpool School of Tropical Medicine.

To effectively neutralize a venom toxin’s surface residues, medical personnel need antivenom specific to the snake that bit the victim. That antivenom may not be accessible, especially in low-resource areas or when the species of the snake is not known by the victim.

India relies on a traditional polyvalent antivenom derived from the so-called big four snakes: the saw-scaled viper, Indian cobra, Russell’s viper, and common krait. But this approach has been found wanting because species outside the big four can have radically different venom proteins, and within each species there can be medically important differences in venom profiles region to region. “You don’t have an antivenom that basically works against them all,” Sunagar says.

For example, Sunagar’s group found that the Sind krait is genetically similar to the common krait of the big four but can produce venom up to 11 times as potent simply because it upregulates certain venom genes and thus produces more copies of certain toxins. This variation renders common antivenoms much less effective against the Sind krait.

Although researchers had known about venom variability for a while, they could not readily quantify or adjust for that variability until the groundbreaking work of Juan José Calvete of the Institute of Biomedicine of Valencia 10–15 years ago. He applied mass spectrometry and protein separation techniques to venom proteomics, or venomics. Calvete’s incorporation of mass spec and an ever-growing database of venom protein profiles make research much easier, says Tan Choo Hock, a venomics researcher at the University of Malaya. He adds that with earlier techniques, just identifying a protein would be a chore.

With ease of analysis, the body of data on venoms has ballooned. As of 2020, there were at least 300 snake venom proteomic studies available, up from around 60 in 2008. And around 40 projects are underway to arrange species’ nucleotide sequences in proper order and document their genomes. Tan says that scientists will need still more genomic analysis if they want to understand these toxins’ actions.

The data contained in these venom profiles are illuminating, but they are also vast. They answer some questions about the contents of venom and raise new ones about how and why snakes developed their arsenals of molecular weapons and defenses.

## Informed by evolution

African spitting cobras display a curious defensive strategy. Although they use venom as other snakes do—injecting it into the bloodstream of their prey—the cobras can also spit venom up to a couple of meters to stave off aggressors.

Casewell’s group found that some spitting cobras overexpress genes that produce phospholipase A2 (PLA2) toxins. These amplify the action of the snakes’ 3FTxs, or three-finger toxins—proteins that usually dominate cobra venoms and whose structures resemble a trio of gnarled, outstretched fingers. When the projectile venom hits its target, the mixture of PLA2s and 3FTxs causes acute pain on contact. When the snake bites, the combined action of the toxins intensifies muscle necrosis.

**Figure:**
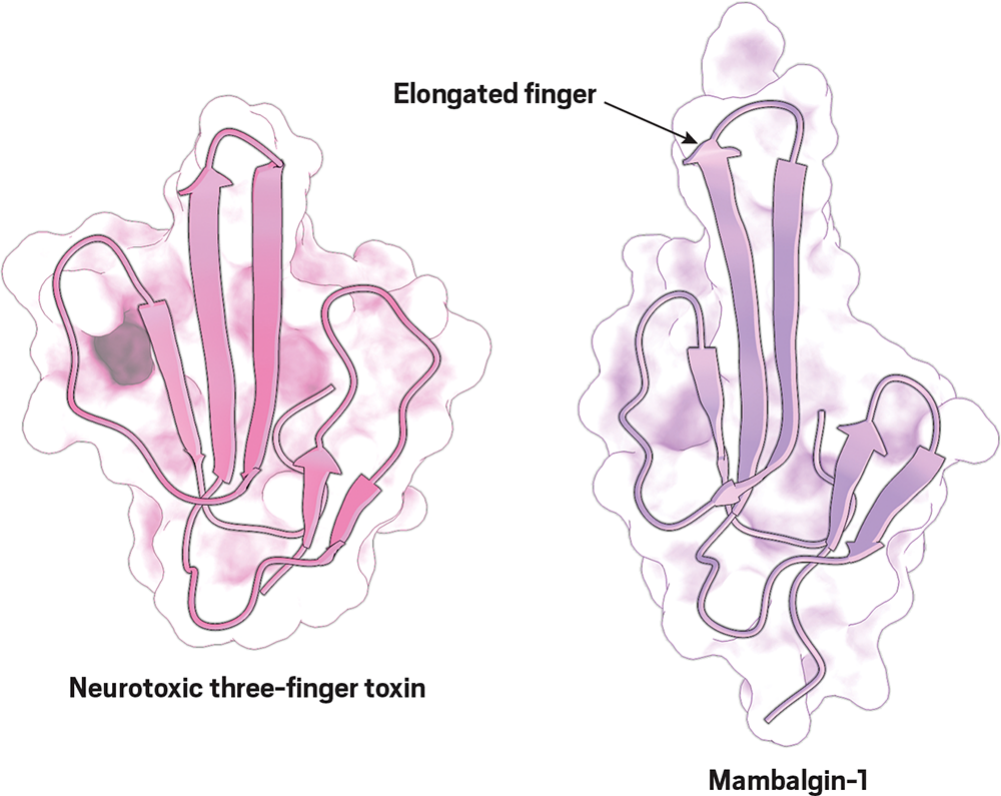
Some researchers say the elongated central finger of mambalgin-1 (right) is the key to the compound’s lack of toxicity and its pain-killing properties. For comparison, three-finger toxins with shorter central fingers, like this one found in the venom of the black-necked spitting cobra, are usually neurotoxic. Credit: Alexandre V. Pinto/University of Porto.

Despite having genes to produce similar toxins, most other cobra species haven’t developed this spitting behavior, and their venoms’ effects are quite different. They are neurotoxins rather than necrotic agents.

Casewell is probing venom adaptations like these because they’re “an example of how understanding the evolution of a snake can inform our understanding of what the venom is doing in a snakebite victim,” he says. That knowledge “hopefully will inform how we can better treat it by blocking specific toxins rather than necessarily the whole venom.”

In a recent preprint—a study that has yet to be peer-reviewed—Casewell and colleagues showed that giving mice a single local injection of the small molecule varespladib reduced the necrotic damage of venoms of spitting cobras. The drug specifically inhibits PLA2s, so its protective effect may occur because the necrotic action of the cobra’s 3FTxs hinges on activation by PLA2s, the researchers write.

“Inhibiting one of these toxins with a targeted drug therapy is sufficient to prevent local tissue damage,” Casewell writes in an email. And it “might therefore be a new ‘evolution informed’ strategy to prevent poor snakebite outcomes in patients bitten by spitting cobras.”

More generally, Pedro Alexandrino Fernandes, a computational chemist at the University of Porto, says that although venom is very diverse, its ingredients can be similar. What often changes is the proportion of each toxin family, but those small differences can cause very significant physiological effects in a snakebite victim. The difference in venom content can exist between closely related species, in which the proportion of toxin families often flip, becoming more or less dominant in a snake’s venom as a species evolves new behaviors.

**Figure:**
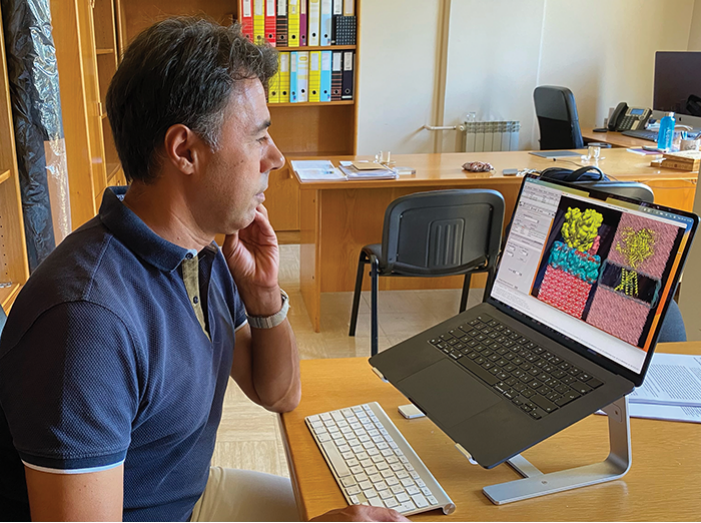
In a pair of simulations, Pedro Alexandrino Fernandes observes how a mambalgin interacts with an acid-sensing ion channel and thus blocks pain signals. Credit: Courtesy of Pedro Alexandrino Fernandes/University of Porto.

As with the spitting cobra, defensive mechanisms in the black mamba have led to unexpected adaptations and a change in its venom makeup. The mamba is notorious for its lethality, but its mambalgins, a family of 3FTx proteins that mambas have retooled, actually reduce pain after the snake bites. Any medicinal chemist would find their soothing power impressive—an analgesic effect on par with morphine. And surprisingly, although the mambalgins exist in a mélange of deadly 3FTxs and dendrotoxins, the mambalgins themselves have evolved to no longer be toxic.

“Why the black mamba has this very strong painkiller, we don’t know,” Fernandes says. “People believe that maybe the black mamba when it injects the venom wants to avoid a counterattack from the prey” because a less painful bite may not be easily noticed.

In 2020, a team of scientists at the University of Science and Technology of China, Tsinghua University, the Chinese Academy of Sciences, and Zhejiang University clarified the structure of mambalgin-1 bound to a human ion channel that plays a role in pain pathways. They found that mambalgin-1 was a new form of 3FTx with an elongated center finger.

At Fernandes’s lab, the researchers are looking to better understand the 3FTx’s mechanism of action and create small molecules that have the same painkilling effect but with certain advantages.

## Exploiting entrenched evolutions

In 2016, when the drug varespladib was initially shown to protect rats against dozens of snake species, small molecules were not the go-to intervention for snakebites. Researchers are still trying to carve out a space in the field for these treatments among traditional ones—usually antibodies extracted from the blood of venom-treated animals.

Perhaps small molecules could be tools for first responders. Because many snakebite deaths occur outside of a clinical setting as a victim is in transit to the hospital, first responders could immediately give a victim a relatively broad-spectrum small-molecule drug and extend the victim’s survival long enough for other care providers to administer antivenom in a clinical setting. In the past decade, as the structures of venom proteins and their interactions have become clearer, an approach involving lab-made molecules has become more feasible.

Researchers have been able to look beyond the vast diversity of amino acids found on toxins’ surfaces and instead look more closely at the proteins’ cores, the internal amino acids that allow the molecule to do its job as a catalyst. Millions of years of evolution have left some of these core sequences more or less untouched within toxin families. So these conserved protein regions present footholds for new treatments—including small molecules—to inhibit otherwise deadly toxins.

For example, many snake venoms’ PLA2 enzymes catalytically break down cell membranes and cause necrosis. That catalytic activity hinges on PLA2s’ calcium ion-binding loop. Varespladib blocks this site, keeping calcium from settling into the protein and deactivating its catalysis.

Similarly, in russellysin—a toxin found in Russell’s viper venom and the most potent coagulation activator known—Fernandes’s team showed computationally how the protein catalyzes its deadly reaction in prey. The researchers focused on three histidines and a glutamic acid that interact with a zinc ion nestled in the protein’s core, all of which are highly conserved across russellysin’s larger toxin family, snake venom metalloproteinases (SVMPs).

Fernandes’s team found that marimastat, a drug that Casewell is investigating to inhibit SVMPs, binds to zinc. The researchers also found that marimastat’s binding conformation bares a striking similarity to the transition state that russellysin’s histidines form around its zinc ion as the toxin wreaks havoc in the bloodstream. Fernandes’s computational insights could lead drug developers to a tweaked version of marimastat that has fewer side effects or is even better at stopping SVMP activity by mimicking these conserved amino acids.

Antibodies still have their place, though. Small-molecule drugs have not yet been shown to work against 3FTxs. These toxins paralyze a menagerie of vertebrates: mammals, birds, reptiles, amphibians, and fish. But 3FTxs get this broad-spectrum toxicity because they maintain a conserved interface that can latch on to an acetylcholine receptor that is common in the neurons of these various animals. That conserved interface might be their Achilles’ heel, however, potentially making the whole family vulnerable to a single broadly neutralizing antibody.

In a recent preprint posted to *bioRxiv*, researchers from the U.S. National Institutes of Health and the San Francisco-based start-up Centivax say they have developed such an antibody. They report that the antibody provided mice with “complete protection” after the researchers injected them with 3FTx-dominant venoms. Combining their antibody treatment with varespladib expanded the types of venoms the mice could endure.

If insights about the long geologic stretch of evolution lead to treatments that keep snakebite victims alive longer, it could not come a minute too soon. Sunagar recalls how, not long ago, he had been looking for a Sind krait in the western Indian state of Rajasthan to collect a venom sample. Unable to find this elusive snake, he returned to his lab in Bengaluru. As soon as Sunagar arrived, he opened a message from a colleague in Rajasthan. It was a photo of a person who’d recently been bitten by that same species.

The victim had not survived. Sunagar had been searching only a few hours away from where the bite happened.

## Virat Markandeya is a freelance contributor to

Chemical & Engineering News, *an independent news outlet of the American Chemical Society*.

